# Understanding Measurements of Vitality in Patients with Chronic Kidney Disease: Connecting a Quality-of-Life Scale to Daily Activities

**DOI:** 10.1371/journal.pone.0040455

**Published:** 2012-07-12

**Authors:** Shunichi Fukuhara, Tadao Akizawa, Satoshi Morita, Yoshiharu Tsubakihara

**Affiliations:** 1 Department of Healthcare Epidemiology, Graduate School of Medicine, Kyoto University, Kyoto, Japan; 2 Division of Nephrology, Department of Medicine, School of Medicine, Showa University, Tokyo, Japan; 3 Department of Biostatistics and Epidemiology, School of Medicine, University Medical Center, Yokohama City University, Yokohama, Japan; 4 Department of Kidney Disease and Hypertension, Osaka General Medical Center, Osaka, Japan; Tehran University of Medical Sciences, Islamic Republic of Iran

## Abstract

**Background:**

Many patients with chronic kidney disease (CKD) suffer from fatigue caused by anemia, but that anemia can be reversed. Successful treatment can be measured as a decrease in fatigue and an increase in energy or vitality, particularly on the vitality (VT) subscale of the SF-36. Changes in VT scores are most commonly interpreted in terms of minimally important differences or standardized effect sizes, but neither a minimally important difference nor a standardized effect size provides information about how patients’ activities are affected. Therefore, we analyzed the association between differences in VT scores and a variable that is meaningful to patients and to society the frequency of going out.

**Study Design:**

Questionnaire survey. Analyses of differences among participants at bseline, and analyses of differences within participants over time.

**Setting and Participants:**

CKD patients who were not on dialysis and were involved in a study of anti-anemia therapy.

**Predictor:**

VT scores.

**Outcome:**

Frequency of going out.

**Measurements:**

VT scores and the frequency of going out.

**Results:**

At baseline, higher VT scores and younger age were associated with going out more often, while sex and the presence of diabetic nephropathy were not associated with the frequency of going out. Greater changes in VT scores over time were associated with greater changes in the frequency of going out, in univariate and multivariate analyses.

**Conclusions:**

At baseline, VT was associated with the frequency of going out. Increases in VT were also associated with increases in the frequency of going out. These results show how VT scores can be linked to daily activities that are important to individual patients and to society.

## Introduction

Anemia causes fatigue in predialysis patients with chronic kidney disease (CKD). That fatigue is alleviated when Hb levels increase after treatment with erythropoiesis-stimulating agents (ESAs). The effects of such treatment are often measured as self-reported decreases in fatigue and increases in energy or vitality. In a recent review of 14 studies, Gandra et al. [1] concluded that ESAs can increase self-reported levels of energy, and a review by Leaf and Goldfarb came to a similar conclusion [2].

In this context, one question is how scores on the vitality (VT) subscale of the SF-36 should be interpreted. Changes in VT scores are most commonly interpreted in terms of minimally important differences [3] or standardized effect sizes [4]. Minimally important differences are cutoff points; they can be used to distinguish treatments that have a pre-specified magnitude of effect from those that do not. Standardized effect sizes such as Cohen’s d express the magnitude of an effect in standard-deviation units and are often interpreted as “small,” “large,” etc. By itself, however, neither a minimally important difference nor a standardized effect size provides information about how differences in vitality affect CKD patients’ lives.

**Table 1:** Demographic and clinical characteristics, and the frequency of going out.

		n (%), or mean ± SD		
Sex				
M		144 (46.6)		
F		165 (53.4)		
Age (years)			64.7±11.8	
<65		138 (44.7)		
≥65		171 (53.3)		
Underlying Disease				
Chronic glomerulonephritis		135 (43.7)		
Diabetic nephropathy		67 (21.7)		
Other		107 (34.6)		
Weight (kg)			56.46±11.06	
Hemoglobin (g/dL)			9.17±0.83	
Ferritin (ng/mL)			128.6±203.8	
TSAT (%)			31.02±12.29	
Serum creatinine (mg/dL)			3.55±1.07	
Ccr (mL/min)			18.79±6.67	
VT			64.1±21.2	
Frequency of going out				
Almost never		31 (10.0)		
1–2 days a week		100 (32.4)		
3–4 days a week		78 (25.2)		
5 or more days a week		100 (32.4)		
Total		309 (100)		

For example, in a recently reported randomized controlled trial, patients with chronic kidney disease were given an ESA, with one of two possible Hb targets [5]. The between-group difference in the change from the baseline value was 4.8 points on the VT scale, which is very close to the minimally important difference of 5 points that was suggested by Bjorner et al. [6]. That difference was also statistically significant (p = 0.025). However, when we ask what that difference means, then the p value of course gives no answer, and the minimally important difference is interpretable only in terms of the population-level phenomena by which it was originally defined.

Interpretability is recognized as an important characteristic of patient-reported outcomes [7], and the impact of anemia treatment on the quality of life of pre-dialysis CKD patients has been studied [8]–[10], but connections between VT scores and daily life are still obscure. Therefore, we analyzed the association between differences in VT scores and a variable that is not generally considered to be a direct manifestation of health-related quality of life, but is nonetheless meaningful to patients and to society: the frequency of going out (we translate the Japanese *gai-shutsu* as “going out”). This paper shows how differences in VT scores, both differences between participants at baseline and differences over time within participants, could be used to predict differences in the frequency of going out.

**Table 2:** Analyses of data collected at baseline: associations of VT score and frequency of going out.

	β	Standard Error	Wald χ^2^	Odds ratio	95% CI	*P*
Model A						
Intercept	−0.87	0.38	–	–	–	–
VT score as continuous variable	0.019	0.01	10.63	1.02	1.01–1.03	0.001
Model B						
Intercept	0.76	0.78	–	–	–	–
VT score as continuous variable	0.02	0.01	10.44	1.02	1.01–1.03	0.001
Age	−0.03	0.01	7.91	0.97	0.95–0.99	0.005
Sex (Male)	0.24	0.26	0.91	1.28	0.77–2.12	0.340
Diabetic nephropathy yes/no (non-diabetic nephropathy)	0.31	0.30	1.05	1.36	0.76–2.44	0.305

## Methods

Data from those patients in Akizawa et al.’s study [5] whose quality-of-life (QOL) questionnaires at baseline and also at 12 weeks after the start of drug administration were complete, without regard to the group to which they were assigned, were analyzed. VT was measured with 4 question items and scored on a scale from 0 to 100, with higher scores reflecting greater vitality [11]. The questionnaires also included one question asking about the frequency of going out. That question had four choices as responses: “I almost never go out”, “I go out one or two days each week”, “I go out three or four days each week”, and “I go out five or more days each week”.

The relationship at baseline between VT score and the frequency of going out was analyzed, as was the relationship between changes in VT scores and changes in the frequency of going out.

This study was approved by the Institutional Review Boards at Ikegami General Hospital, the Medical Corporation of Showakai, and Kochi Takasu Hospital. All participants giave written informed consent.

### Statistical Analysis

#### Analyses of data collected at baseline

To analyze the relationship at baseline between VT score and the frequency of going out, two logistic-regression models were used. In both models, the dependent variable was the baseline frequency of going out, which had been dichotomized into low frequency (fewer than three times each week) and high frequency (three or more times each week).

In the simple model (model A), there was only one independent variable: the baseline VT score. In the more complex model (model B), sex, age, and the presence or absence of diabetic nephropathy were included as covariates.

**Table 3:** Associations between the change in VT scores and the improvement in the frequency of going out (VT change score as the independent variable).

	β	Standard Error	Wald χ^2^	Odds ratio	95%CI	*P*
Model C1						
Intercept	−1.23	0.14	–	–	–	–
VT change score (Continuous)	0.03	0.01	13.80	1.03	1.01–1.05	<0.001
Model D1 (covariates: age, sex, diabetic nephropathy)						
Intercept	−2.28	0.83	–	–	–	–
VT change score (Continuous)	0.02	0.01	14.26	1.03	1.01–1.05	<0.001
Age	0.02	0.01	1.95	1.02	0.99–1.04	0.162
Sex (Male)	−0.09	0.29	0.09	0.92	0.52–1.63	0.769
Diabetic nephropathyyes/no (non-diabeticnephropathy)	−0.06	0.34	0.03	0.94	0.48–1.84	0.855
Model E1: Same as Model D1 except patients at the floor at baseline were excluded to avoid regression to the mean						
Intercept	−2.17	0.91	–	–	–	
VT change score (Continuous)	0.02	0.01	7.65	1.02	1.01–1.04	0.005
Age	0.02	0.01	1.25	1.02	0.99–1.04	0.263
Sex (Male)	−0.30	0.33	0.86	0.74	0.39–1.40	0.354
Diabetic nephropathyyes/no (non-diabeticnephropathy)	−0.11	0.39	0.08	0.90	0.42–1.92	0.783

#### Analyses of changes over time

To model the association between changes in VT scores and changes in the frequency of going out, logistic regression was again used.

There were two independent variables: one was the change in VT score, and the other was that change expressed as a standardized effect size and then categorized (SES) [4]. The SES of the change in VT score was computed as follows: (individual score at 12 weeks – individual score at baseline)/standard deviation of the scores at baseline.

**Figure 1:**
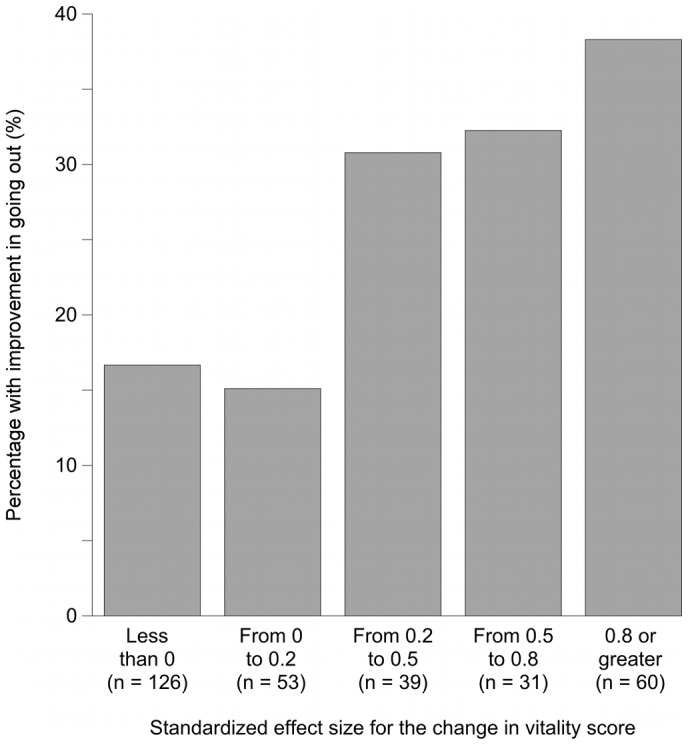
Relationship between the SES categories of changes in VT scores and the proportion of participants who had improvement in the frequency of going out. Comparing the group with the greatest increase in VT score to the group with a decrease and to the group with almost no increase, the Figure shows that more than twice as many people in the former group reported improvement in their frequency of going out. That is, groups with greater increases in VT scores had more peole who reported increases in the frequency of going out.

**Figure 2:**
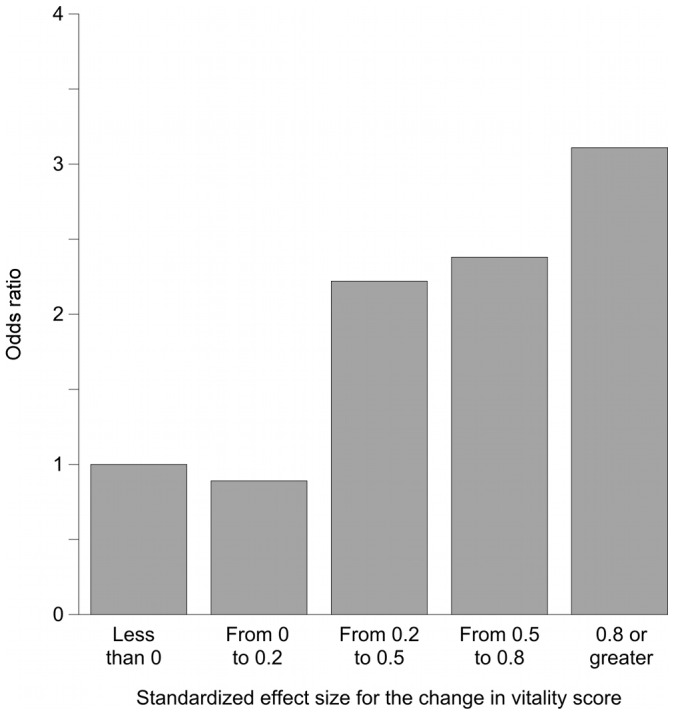
Association between changes in VT scores and changes in the frequency of going out. The independent variable is the categories of SES (SES of “<0” as the reference standard). This Figure shows the results from model C2, in which the change in VT score was the only independent variable. Greater changes in VT scores were clearly associated with going out more frequently.

**Figure 3:**
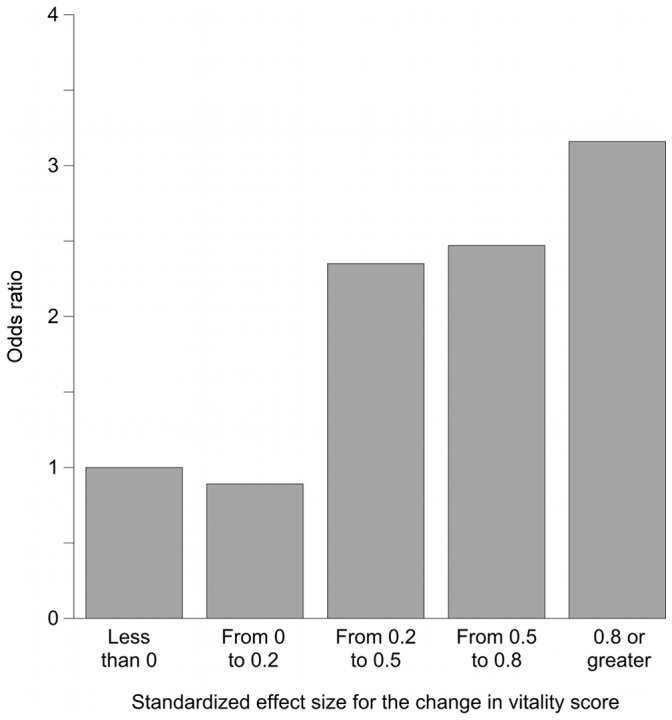
Association between changes in VT scores and changes in the frequency of going out. The independent variable is the categories of SES (SES of “<0” as the reference standard). This Figure shows the results from model D2, which included age, sex, and the presence or absence of diabetic nephropathy as covariates. The odds ratios are almost exactly the same as those shown in Figure 2, which indicates that the association of increases in VT scores with going out more frequently was robust even after likely confounders were accounted for.

**Figure 4:**
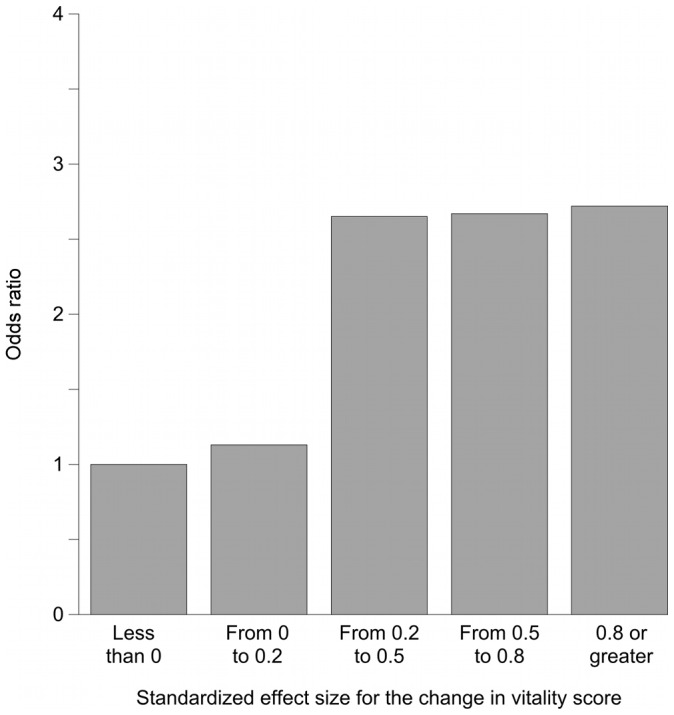
Association between changes in VT scores and changes in the frequency of going out. The independent variable is the categories of SES (SES of “<0” as the reference standard). This Figure shows the results from model E2, which was same as Model D2 except that patients whose values were at the floor at baseline were excluded. Compared with the results of model E2 (Figure 3), the most noteworthy difference is that the magnitude of the association was somewhat attenuated in the highest of the four categories of VT increase. We interpret this result as indicating that the strong association between increases in VT scores and increases in the frequency of going out was not caused by regression to the mean.

SES results are often interpreted in terms of categories, and for this study five categories of SES were defined. The values most commonly used as “borders” between SES categories are 0.2, 0.5, and 0.8 [4]. Using those three criteria, we defined the highest category as “greater than or equal to 0.8”, the next lower category as “from 0.5 to 0.8”, and the next lower category as “from 0.2 to 0.5”. SES values less than 0.2 include both very small positive effects and also all negative effects. Therefore, to separate the positive effects from the negative effects, we defined the two lowest categories as “from 0 to 0.2” and “less than 0”. This gave a total of five categories.

The dependent variable was the change in the frequency of going out, after it had been dichotomized into “increase” and “no increase”.

Two simple models, each of which had only one independent variable (either the change in VT score (model C1) or the category of SES (model C2)), were tested. More complex models were also used, in which sex, age, and the presence or absence of diabetic nephropathy were included as covariates (models D1 and D2). Finally, the more complex models were used again, but after exclusion of data from the participants who at baseline were in the lowest category of going out (models E1 and E2). They were excluded because of concern that increases from the lowest category might have been caused by regression toward the mean.

For all analyses, alpha = 0.05 (two-sided), and 95% confidence intervals are shown.

## Results

Usable data were obtained from 144 men and 165 women (Table 1).

### Data Collected at Baseline

At baseline (Table 2), higher VT scores were associated with going out more often. Age was also associated with going out, but sex and diabetic nephropathy were not.

### Changes Over Time

Greater changes in VT scores were associated with greater changes in the frequency of going out (Table 3), with both the univariate model (model C1) and the multivariate models (models D1 and E1). In the multivariate models, none of the covariates had a p value less than 0.05.

Figure 1 shows, for each SES category, the proportion of respondents in that category whose frequency of going out increased. The frequency of going out increased in approximately 15% of those whose SES was less than 0.2, and it increased in more than 30% of those whose SES was greater than or equal to 0.2. In these data, an SES of 0.2 was equal to a change in VT score of 4.2 points.

Figures 2, 3, and 4 show odds ratios and adjusted odds ratios for increases in the frequency of going out. The SES category of “less than zero” was the reference category. For both the univariate and the multivariate models, the patterns are similar to that shown in Figure 1. None of the covariates in models C2, D2, or E2 had a p value less than 0.05.

## Discussion

The results of the analyses of the baseline data show that VT was associated with the frequency of going out, and the results of the analyses of changes over time show that increases in VT were associated with increases in the frequency of going out.

We expect that documenting such associations will be particularly important for patient-centered clinical practice, in which it is necessary to “translate” risk reductions, numbers needed to treat, and QOL scores into more concrete terms to help patients understand their meaning and also to give healthcare workers insights into patients’ lives outside the context of the clinical encounter.

Reports of QOL scores have become common in many clinical fields, including clinical nephrology [8], but such reports do not often give clear ideas about how the scores can be interpreted. For example, patients may be told that treatment of their anemia can be expected to increase their VT score by about 5 points (as found by Akizawa [5]), but of course that alone is not very informative. It raises the question “What does a 5-point increase mean?” Some previous studies have approached such questions via the concept of the minimally important difference. For example, Bjorner et al. [6] quantified the effects of various medical conditions on VT scores, as well as the association between VT scores and life events such as job loss, hospitalization, and death. On the basis of their results, they proposed that 5 points be used as the minimally important difference for groups with below-average VT scores. Applying that criterion, we might expect treatment of anemia to result in a group-level difference in VT scores that would be important.

Another common approach to QOL scores involves expressing differences in standard-deviation units. As noted by Norman et al. [12], minimally detectable differences are often about 0.5 SDs. In this study, a VT score difference of about 5 was within the 0.2 to 0.4 range of standardized effect size that is usually called “medium” or “moderate” [2].

Nonetheless, whether we focus on minimally important differences (quantified by disease and by life events) or on thresholds in standard-deviation units, the implications from a patient’s perspective can still be vague. Thus, attention should also be paid to the link between VT scores and effect indicators that are more immediately understandable to patients and more directly relevant to social policies. We examined the frequency of *gai-shutsu* as one such effect indicator.

Interpretation of the present results therefore depends greatly on the meaning of the term *gai-shutsu*. While we translate it into English as “going out”, it should be kept in mind that *gai-shutsu* is generally not used to describe any and all instances of leaving one’s home. Instead, it is generally used to describe instances of leaving either one’s home or one’s workplace for a non-obligatory activity. Thus, it generally does not include going to work or to school. It might also not include going to a clinic or a hospital for a pre-scheduled outpatient appointment. In such a situation one would probably not use the general term *gai-shutsu*, but would instead mention the destination explicitly. *Gai-shutsu* can include going out alone, but it also covers many activities done in groups.

With that meaning in mind, these results provide a basis for seeing changes in VT scores as indicators of patients’ daily lives. In particular, among older people in Japan and in Japanese society generally, social isolation and the loosening of family and community ties are becoming important problems. The proportion of elderly people living alone increased from 4.3% for men and 11.2% for women in 1980 to 9.7% for men and 19.0% for women in 2005 [13].

Any resulting tendency toward social isolation might be alleviated by interventions promoting activities outside the home. It is reasonable to expect that unemployed elderly people who go out (in the sense of *gai-shutsu*, as described above) are less likely to burden their family or social-service agencies. Increases in vitality might provide a foundation for increasing *gai-shutsu*, which would be both individually and socially beneficial. Another implication of the present work is that such improvements might be expected from medical interventions, and specifically from the treatment of anemia [2].

One important caveat derives from the fact that this study was a type of secondary analysis. The data were originally collected as part of a randomized trial of two different targets for anti-anemia therapy, and the opportunity to use them for the present purpose was incidental. Therefore these results must not be taken as definitive and conclusive. Because future confirmatory work is still required, these results can instead be seen as suggestive of a possibly important association. They can also be used to guide the design of future prospective research.

Such prospective studies are realistic and they should be done. Going out is not very commonly measured, but use of the SF-36 is now well-established in Japan, and it is widely used in contexts ranging from in-hospital RCTs to community-based observational studies [14], [15]. The many ongoing and planned uses of the SF-36 will result in VT scores being recorded, and plans can be made prospectively to use those scores as sources of information about how often people go out. Plans can also be made to include, as an important part of future studies, measurements of the frequency of going out. If the results of the present study are confirmed, then information about connections between VT and *gai-shutsu* might expand the possibilities for interpreting the results of a wide range of clinical interventions and social-welfare programs. Asking about the frequency of *gai-shutsu* is of course not the only way to explore the meaning of VT scores, and we hope that other work will continue putting clinical measurements into concrete and easily understood terms.
